# Construction of a prediction model for pneumonia in children undergoing neurosurgery based on regular clinical laboratory tests and baseline information

**DOI:** 10.3389/fped.2025.1638012

**Published:** 2025-08-06

**Authors:** Shumei Zhang, Hongyao Wang, Shuting Lin, Yihuang Zhang, Yingbin Lin, Wenhua Fang, Yue Chen

**Affiliations:** ^1^ Department of Neurosurgery, National Regional Medical Center, Binhai Campus of the First Affiliated Hospital, Fujian Medical University, Fuzhou, China; ^2^ Department of Neurosurgery, The First Affiliated Hospital, Fujian Medical University, Fuzhou, China; ^3^ Neurosurgery Research Institute, Fujian Provincial Institutes of Brain Disorders and Brain Sciences, The First Affiliated Hospital, Fujian Medical University, Fuzhou, Fujian, China

**Keywords:** neurosurgery, children, pulmonary infection, risk factor analysis, prediction model establishment

## Abstract

**Objectives:**

Pneumonia is a common complication in children undergoing neurosurgery, leading to prolonged length of stay as well as increased hospital expenses. A prediction model for pneumonia in children undergoing neurosurgery based on common laboratory indicators is an effective clinical measure for early intervention in high-risk patients. In this study, we proposed to construct a pneumonia prediction model for children undergoing neurosurgery by selecting routine baseline characteristics and laboratory indicators.

**Methods:**

This study retrospectively collected children admitted from January 2021 to April 2025. The data collected included common clinical baseline data and regular laboratory test results. Variables were filtered by multivariate regression and constructed a prediction model.

**Results:**

Screening revealed that whether emergency admission, whether surgical treatment, type of disease, serum creatinine level and neutrophil count were statistically different indicators. A prediction model was constructed based on the above indicators, and the C-statistic values of the model were 0.835 (test set, 95% CI: 0.7692–0.9006) and 0.716 (validation set, 95% CI: 0.5803–0.8509), which were satisfactory. And a clinically usable nomogram based on the above model was constructed.

**Conclusions:**

Hospital-acquired pneumonia is a common complication in children undergoing neurosurgery and may be related to a variety of factors. Using basic clinical baseline data and laboratory data to monitor and detect high-risk patients in the early stages of the disease is a useful clinical attempt that deserves further exploration.

## 1 Introduction

Pneumonia has been a serious complication troubling neurosurgeons, especially in children undergoing neurosurgery. Pneumonia increases the hospitalization time of patients, causing increased morbidity and mortality, long-term persistent sequelae, and increased healthcare costs, especially for hospitalized neurosurgical patients (1, 2). The many factors that contribute to pneumonia make it generally difficult to predict pneumonia with existing clinical indicators alone, and the addition of novel clinical tests leads to increased costs and unnecessary costs.

Therefore, constructing a predictive model for pneumonia by routine clinical test indicators and establishing a predictive model for pneumonia are important approaches to improve the diagnosis of pneumonia in children undergoing neurosurgical procedures and to improve the prognosis of patients (3, 4). Neutrophil/lymphocyte ratio, lymphocyte/monocyte ratio, and other peripheral blood inflammatory markers are a series of emerging laboratory indicators that have been shown to correlate with the clinical prognosis of a variety of diseases, such as infections and neoplasms; however, there is a lack of research on the relationship between the above indicators and pneumonia in children undergoing neurosurgical procedures (5, 6). In this study, we analyzed the factors associated with pneumonia in neurosurgical inpatients, filtered the appropriate indicators, and constructed a prediction model for pneumonia in neurosurgical inpatients.

## 2 Methods

Retrospective clinical data were collected from January 2021 to April 2025 on 267 children undergoing neurosurgery in the Department of Neurosurgery of the First Affiliated Hospital of Fujian Medical University. We designed this study under the guidelines outlined in the Declaration of Helsinki and approved by the local Ethics Committee of the First Affiliated Hospital of Fujian Medical University (Fujian, China).

## 3 Study population

We obtained informed consent from the patient or their authorized legal representative if patients cannot sign the form by themselves. Inclusion criteria: patients aged <18 years who received inpatient surgical/non-surgical treatment. Exclusion criteria: (1) patients diagnosed with pneumonia or other systemic infections within 48 hours of admission; (2) patients who failed to undergo laboratory tests within 48 hours of admission; and (3) patients using immunosuppressive drugs or receiving immunosuppressive therapy.

## 4 Patients management

Hospital-acquired pneumonia refers to pneumonia acquired after 48 hours of admission to the hospital. The preoperative prophylactic antibiotic protocol follows our antibiotic stewardship committee's recommendation to use 1 gram of cefazolin half an hour before surgery. If the procedure is more than 3 hours, use the same dose of cefazolin again.

## 5 Data collection

We investigated the relationship between the parameters collected by pneumonia. The baseline conditions of the patients were collected, as well as the results of routine peripheral blood tests in the laboratory, including routine blood tests, biochemical tests. Laboratory indicators are collected within 48 hours of admission, and patient laboratory data are collected through the medical record system.

Based on the peripheral blood test results, the values of relevant peripheral blood inflammatory marker parameters were calculated as follows: neutrophil/lymphocyte ratio (NLR) as the number of neutrophils divided by the number of lymphocytes, platelet/lymphocyte ratio (PLR) as the number of platelets divided by the number of lymphocytes, lymphocyte/monocyte ratio (LMR) as the number of lymphocytes divided by the number of monocytes, platelet/center granulocyte ratio (PNR) is the platelet count divided by the neutrophil count, platelet/albumin ratio (PAR) is the platelet count divided by the albumin content, and systemic inflammation response index (SIRI) is the neutrophil count multiplied by the monocyte count divided by the lymphocyte count (7–10).

## 6 Statistical analysis

R (version 4.5.0) was applied for data analysis. For statistical results of categorical variables, expressed as counts (percentages), the *χ*2 test or Fisher exact test was used. For ranked variables, the Kruskal–Wallis Test rank sum test was used. Continuous variables other than peripheral blood inflammatory marker parameter values were discretized and converted to categorical or hierarchical variables according to clinical practice. Variables with single-variable relationships (*P* < 0.1) with pneumonia were screened among the candidate variables, and the above selected variables were included in the multivariable linear regression analysis, and variable elimination was performed using stepwise backward and stepwise forward methods to obtain the final variables (*P* < 0.05). Pneumonia prediction model was constructed using the final variables obtained above. Further evaluation was done using C-index to assess the discriminative power of the model, calibration plots were used to assess the fit of the model, and internal verification was done using bootstrap (bootstrap). Decision analysis curves were constructed to further assess the benefit of the model for clinical decision making (calibrated to the reported 10% incidence of pneumonia), clinical impact curves were constructed to model the effect in clinical diagnosis, and clinically usable nomograms were created.

## 7 Results

The primary analysis included as many general clinical indicators related to pneumonia as possible. In the data preprocessing stage, some continuous data were discretized according to the clinical reference value interval and classified into three levels: “low”, ‘normal’ and “ high”. For indicators such as NLR and LMR, since the distribution of these indicators in this group is not yet clear, direct discrete transformation lacks a valid basis, so they continue to be analyzed in the form of continuous variables. Since some patients were admitted to the hospital with imaging results from other hospitals and some elective patients had their imaging examinations perfected at their appointments after 48 hours, imaging examinations were not included in the collection due to standardization and timing issues.

**Figure 1:**
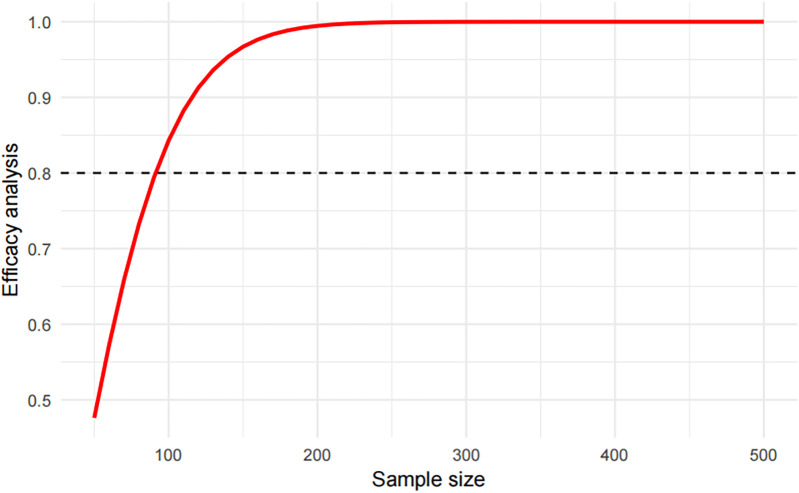
Multivariate regression efficacy analysis.

The distribution of the above indicators among the groups is shown in Table 1. The data were divided into a test set and a validation set, and variables with a univariate relationship (*P* < 0.1) with pneumonia were screened in the test set and included in the multivariate linear regression analysis. Stepwise backward and stepwise forward multivariate logistic regression analyses showed that emergency admission or not, surgical treatment or not, type of disease, serum creatinine level, and neutrophil count were statistically different indicators (Table 2). Evaluation based on analytic efficacy showed that the sample size included in this study at the medium effect level met statistical requirements (Figure 1).

**Table 1:** The comparison of baseline data between the HAP and the non-HAP.

Object	All (*n* = 267)	Non-HAP (*n* = 222)	HAP (*n* = 45)	*p*-value
Demographics				
Sex				0.712
Female, *n* (%)	115 (43.1%)	94 (42.3%)	21 (46.7%)	
Male, *n* (%)	152 (56.9%)	128 (57.7%)	24 (53.3%)	
Age, median (IQR)	11 (8–14)	11 (8–15)	10 (5–13)	0.007
Clinical features				
Height, median (IQR)	148 (129–163)	148 (134–165)	140 (113–160)	0.037
Weight, median (IQR)	40.0 (27.5–54.0)	40.5 (29.0–53.8)	37.0 (19.0–56.0)	0.108
T, median (IQR)	36.5 (36.5–36.7)	36.5 (36.5–36.7)	36.5 (36.5–36.8)	0.965
Emergency admission				0.004
No, *n* (%)	256 (95.9%)	217 (97.7%)	39 (86.7%)	
Yes, *n* (%)	11 (4.12%)	5 (2.25%)	6 (13.3%)	
Surgery				< 0.001
No, *n* (%)	100 (37.5%)	97 (43.7%)	3 (6.67%)	
Yes, *n* (%)	167 (62.5%)	125 (56.3%)	42 (93.3%)	
Primary diagnosis				0.094
Trauma, *n* (%)	9 (3.37%)	6 (2.70%)	3 (6.67%)	
Spontaneous hemorrhage, *n* (%)	9 (3.37%)	8 (3.60%)	1 (2.22%)	
Neoplasms, *n* (%)	100 (37.5%)	78 (35.1%)	22 (48.9%)	
Other, *n* (%)	149 (55.8%)	130 (58.6%)	19 (42.2%)	
Quarter of admission				0.037
Q1, *n* (%)	68 (25.5%)	58 (26.1%)	10 (22.2%)	
Q2, *n* (%)	40 (15.0%)	34 (15.3%)	6 (13.3%)	
Q3, *n* (%)	96 (36.0%)	85 (38.3%)	11 (24.4%)	
Q4, *n* (%)	63 (23.6%)	45 (20.3%)	18 (40.0%)	
LOS, median (IQR)	13.0 (8.00–21.5)	11.0 (7.25–17.0)	22.0 (13.0–32.0)	< 0.001
Laboratory data				
WBC				0.001
Normal, *n* (%)	104 (39.0%)	95 (42.8%)	9 (20.0%)	
Low, *n* (%)	133 (49.8%)	108 (48.6%)	25 (55.6%)	
High, *n* (%)	30 (11.2%)	19 (8.56%)	11 (24.4%)	
RBC				0.412
Normal, *n* (%)	139 (52.1%)	119 (53.6%)	20 (44.4%)	
Low, *n* (%)	16 (5.99%)	14 (6.31%)	2 (4.44%)	
High, *n* (%)	112 (41.9%)	89 (40.1%)	23 (51.1%)	
PLT				0.087
Normal, *n* (%)	174 (65.2%)	150 (67.6%)	24 (53.3%)	
Low, *n* (%)	2 (0.75%)	1 (0.45%)	1 (2.22%)	
High, *n* (%)	91 (34.1%)	71 (32.0%)	20 (44.4%)	
Neut, median (IQR)	3.26 (2.50–4.22)	3.24 (2.46–4.04)	3.72 (2.81–5.15)	0.018
Lymph, median (IQR)	2.33 (1.94–3.01)	2.30 (1.95–3.00)	2.63 (1.85–3.32)	0.565
Mono, median (IQR)	0.37 (0.30–0.47)	0.37 (0.30–0.48)	0.39 (0.30–0.45)	0.881
NLR, median (IQR)	1.34 (0.96–1.78)	1.31 (0.96–1.77)	1.45 (0.98–2.02)	0.310
PLR, median (IQR)	111 (89.1–139)	110 (89.1–138)	119 (91.3–171)	0.353
LMR, median (IQR)	6.52 (4.74–8.79)	6.46 (4.76–8.49)	7.00 (4.55–9.82)	0.444
PNR, median (IQR)	84.3 (62.2–109)	84.2 (63.9–110)	86.4 (47.9–106)	0.284
SIRI, median (IQR)	0.51 (0.30–0.82)	0.51 (0.29–0.80)	0.51 (0.35–1.00)	0.419
LDH, median (IQR)	202 (172–234)	200 (165–229)	227 (193–258)	0.001
ALB, median (IQR)	44.9 (42.9–47.1)	44.8 (43.0–47.1)	45.2 (42.9–47.5)	0.815
Tbil, median (IQR)	7.10 (5.40–9.50)	7.10 (5.40–9.57)	7.10 (4.90–9.20)	0.597
ALT, median (IQR)	11.0 (8.00–17.0)	11.0 (8.00–17.0)	13.0 (10.0–18.0)	0.114
Cr, median (IQR)	47.0 (37.0–60.0)	48.0 (38.0–61.0)	43.0 (34.0–52.0)	0.007
Urea, median (IQR)	4.01 (3.30–4.79)	4.00 (3.31–4.86)	4.04 (3.13–4.30)	0.165
K^+^, median (IQR)	4.43 (4.18–4.68)	4.42 (4.18–4.66)	4.53 (4.19–4.81)	0.144
Na^+^, median (IQR)	140 (139–142)	140 (139–142)	141 (139–141)	0.319

Values are reported as number, number (%), median (25%–75%), and mean ± SD. HAP, hospital-acquired pneumonia; non-HAP, non-hospital-acquired pneumonia; T, temperature; LOS, length of stay; WBC, white blood cell; Neut, neutrophil; Lymph, lymphocyte; Mono, monocyte; RBC, red blood cell; Hb, hemoglobin; PLT, platelet; NLR, neutrophil-to-lymphocyte ratio; PLR, platelet-to-lymphocyte ratio; LMR, lymphocyte-monocyte ratio; PNR, neutrophil—red blood cell ratio; SIRI, systemic inflammatory response index; LDH, lactate dehydrogenase; ALB, albumin; Tbil, total bilirubin; ALT, alanine aminotransferase; Cr, creatinine; K+, potassium; Na+, sodium.

**Table 2:** Multivariate model analysis of possible predictors of POP.

Parameter	Univariable analysis			Multivariable analysis		
	OR	97.50%CI	*P*	OR	97.50%CI	*P*
Age		0.084–3.134	0.470			
Height	0.999	0.968–1.031	0.936			
Emergency admission	23.023	0.370–3,975.322	0.208	28.596	1.364–2,206.445	0.076
Surgery	18.243	4.315–144.063	0.001	14.930	3.858–105.874	0.001
Primary diagnosis						
Spontaneous hemorrhage	0.004	0.000–0.463	0.079	0.010	0.000–0.677	0.068
Neoplasms	1.093	0.118–13.591	0.940	1.438	0.195–13.663	0.727
Other	0.329	0.034–3.928	0.342	0.534	0.071–5.028	0.549
Season						
Q2	0.356	0.062–1.790	0.222			
Q3	0.519	0.133–1.991	0.335			
Q4	1.036	0.278–3.879	0.957			
LDH	1.000	0.992–1.005	0.949			
Cr	0.976	0.926–1.028	0.363	0.962	0.927–0.994	0.026
Urea	1.075	0.656–1.768	0.774			
Neut	1.121	0.643–1.875	0.670	1.242	0.997–1.536	0.044
WBC						
Low	1.136	0.273–5.022	0.862			
High	3.780	0.500–29.173	0.192			
PLT						
High	1.008	0.344–2.852	0.989			
NLR	1.058	0.717–1.782	0.812			

LDH, lactate dehydrogenase; Cr, creatinine; WBC, white blood cell; Neut, neutrophil; PLT, platelet; NLR, neutrophil-to-lymphocyte ratio; PLR, platelet-to-lymphocyte ratio.

**Figure 2:**
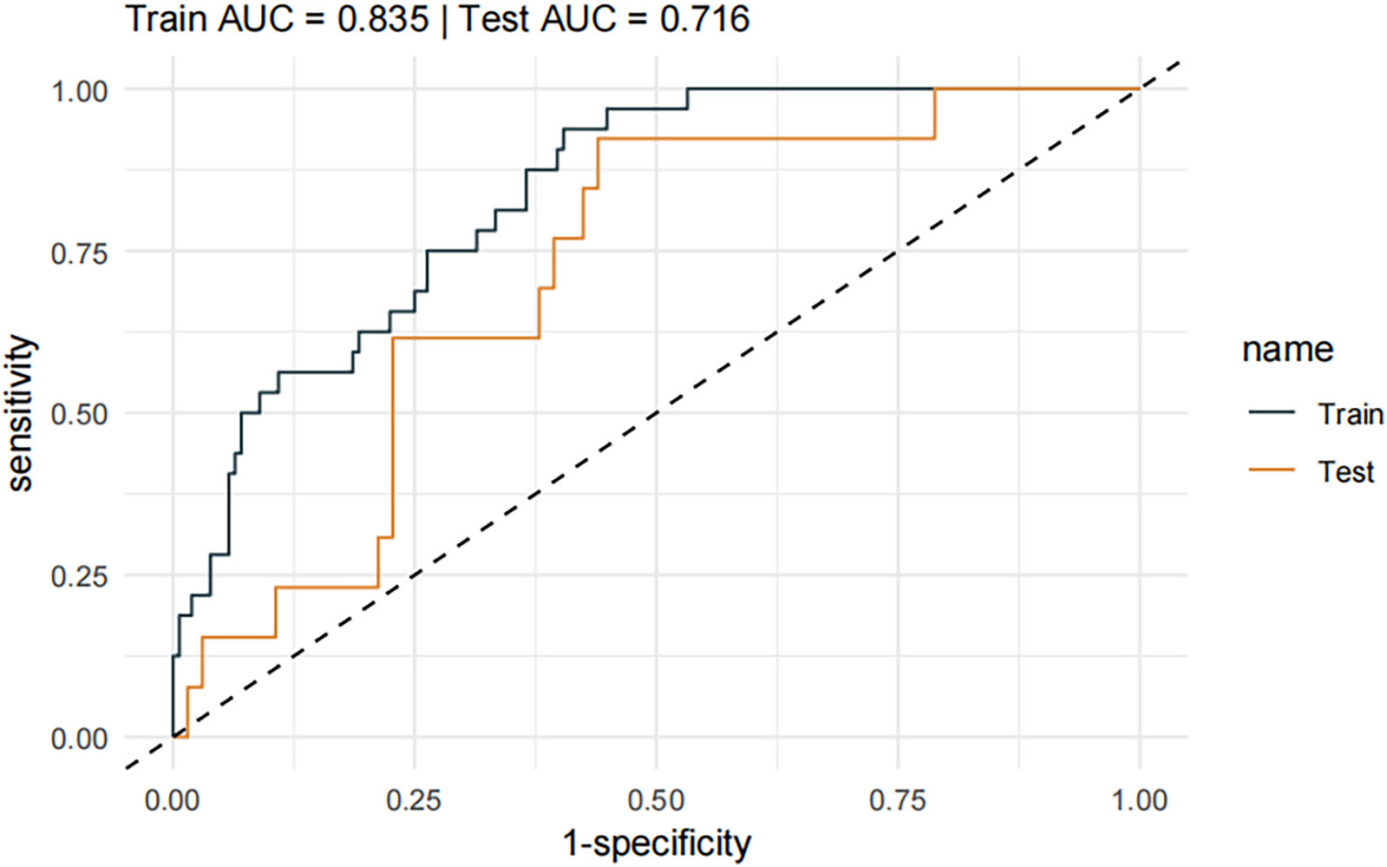
Receiver operating characteristic curves of the training and testing sets.

**Figure 3:**
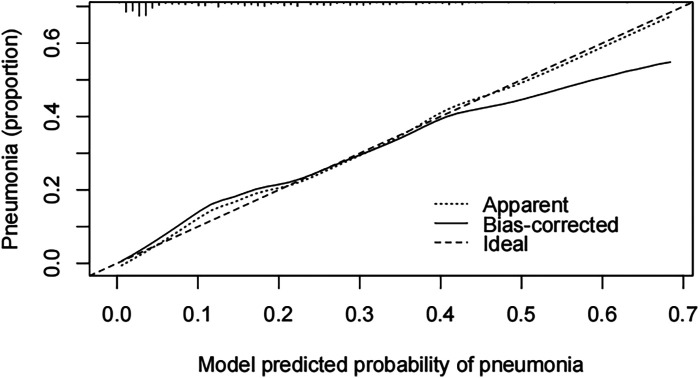
Calibration between predicted and observed outcomes.

**Figure 4:**
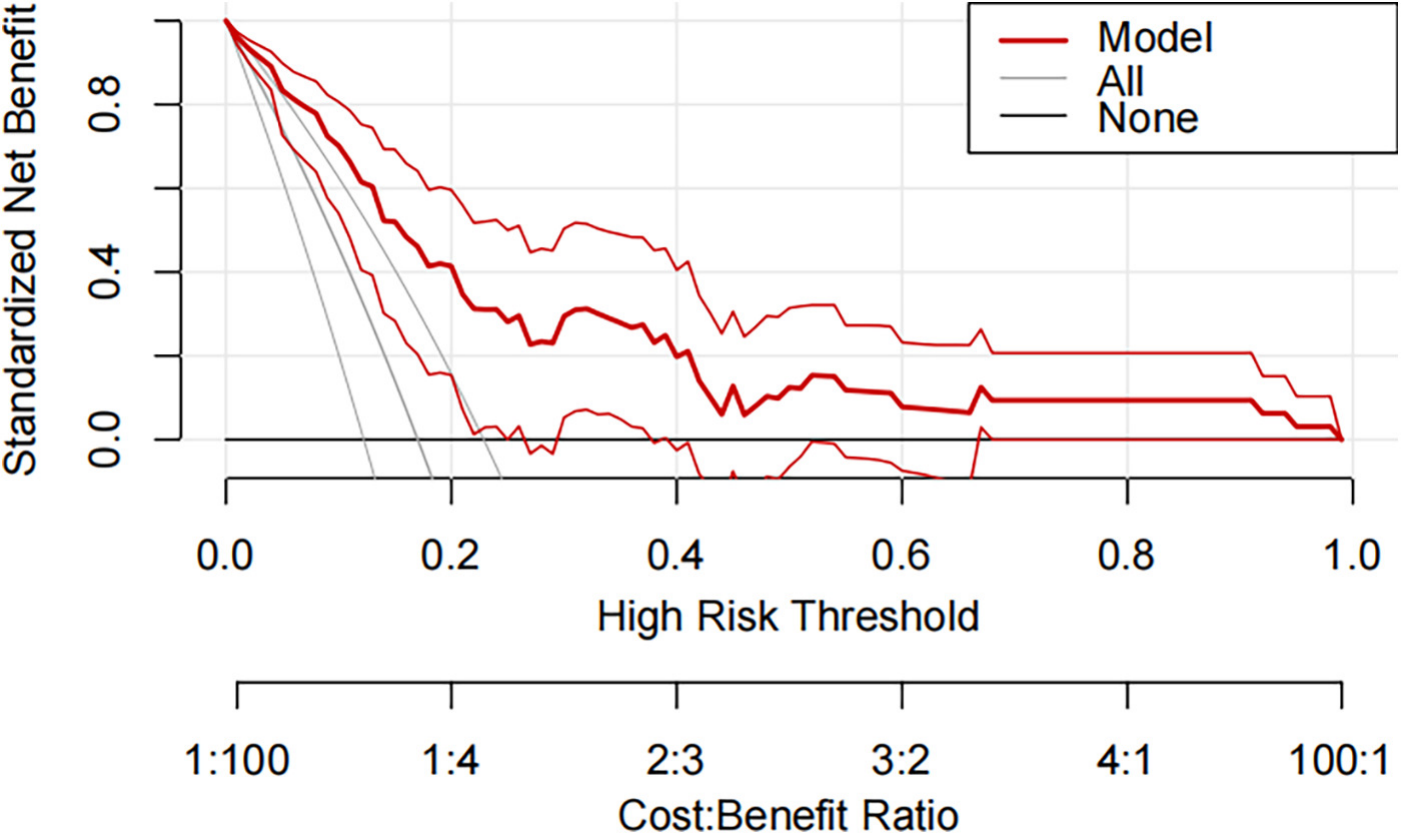
Decision curve analysis for predicting the incidence of pneumonia during hospitalization in pediatric neurosurgical patients in the training and testing sets.

**Figure 5:**
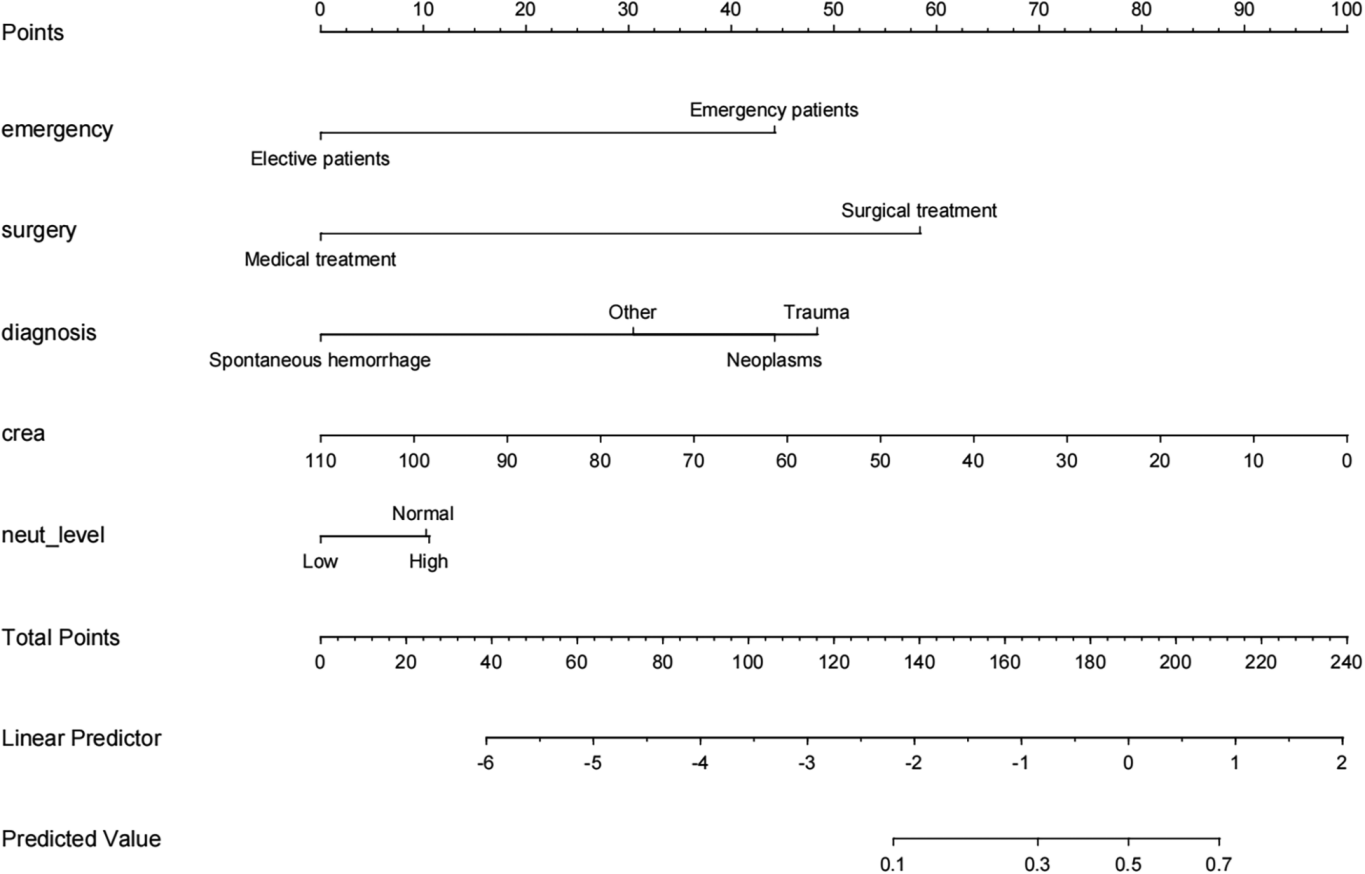
Nomogram for predicting the incidence of pneumonia during pediatric hospitalization.

A pneumonia prediction model was constructed using the above five indicators of “emergency admission, surgical treatment, disease type, creatinine level, and neutrophil count”, C-statistics were calculated, and the model's discriminatory power was further evaluated by sensitivity, specificity, and Youden's index (Figure 2). The C-statistic value of the model in the test set was 0.835 (95% CI: 0.7692–0.9006), with a sensitivity of 93.8%, a specificity of 59.6%, and a Youden's index of 0.107. The validity of the above model was tested in the validation set, in which the C-statistic value of the model was 0.716 (95% CI: 0.5803–0.8509), with a sensitivity of 92.3% and a specificity of 59.6%. sensitivity of 92.3%, specificity of 56.1% and Youden index of 0.137. The accuracy of the model in predicting pneumonia was described using calibration curves and validated using the bootstrap (bootstrap) method (Figure 3). The fit evaluated using mean squared error, the model had a mean squared error of 0.00051 with no overfitting. The possible clinical benefits of the model were evaluated by building a decision model, and the model was able to demonstrate a diagnostic advantage in the range of 10% to 30% risk of pneumonia (Figure 4). To facilitate clinical interpretation, a nomograms of clinical pneumonia prediction based on the above model was further developed (Figure 5).

## 8 Discussion

Pneumonia is a common complication in children undergoing neurosurgery. Several clinical studies have attempted to screen for predictors of pneumonia, but there is still a lacking of widely accepted and used predictors of pneumonia. Introducing new laboratory tests to predict pneumonia may limit potential for further clinical translation; therefore, it is important to develop predictive models for pneumonia based on routine clinical indicators. In this study, we analyzed the relationship between commonly used clinical indicators and pneumonia in children undergoing neurosurgery, filtered out multiple variable factors, combined the above factors to construct a prediction model for pneumonia in children undergoing neurosurgery, and found that the model has good predictive ability for pneumonia in children undergoing neurosurgery, which is potentially clinically significant.

This study included 267 children undergoing neurosurgery with a pneumonia infection rate of 16.85%. A multifactorial model was constructed with relevant indicators screened by univariate analysis, and the C-statistic values of the model were 0.835 (test set) and 0.716 (validation set), suggesting that the model had a good predictive effect. Further evaluation and analysis of the model showed that the multifactor model was not overfitted. Further evaluation of the diagnostic effectiveness of the model showed that the model demonstrated diagnostic advantages in the range of 10% to 30% risk of pneumonia, which is consistent with real clinical scenarios of use. Consistency analysis showed that the multifactorial model was in good agreement with clinical observations. Based on the above model evaluation results, the clinically available diagnostic tools were mapped through the form of nomograms.

The relationship between the variables included in this model and pneumonia may be linked in the following ways. First, the type of admission of the patient is obvious for prognosis; the majority of patients admitted in the emergency setting have a combination of acute and critical neurosurgical conditions, and are admitted in worse general condition, in a susceptible state, and prone to pneumonia (11–13). Second, neurosurgical procedures are usually performed under general anesthesia, and patients undergoing general anesthesia and mechanically assisted ventilation are significantly more likely to develop pneumonia (14–16). In terms of the type of disease, spontaneous cerebral hemorrhage is relatively rare in pediatric patients (3.37% in this case), with less bleeding and less impact on the systemic condition. On the other hand, although trauma is also relatively rare in pediatric patients (3.37% in this case), simple head trauma is rare and is often a part of the systemic injury, and therefore has a greater impact on the systemic condition and is more severe. In contrast, patients with brain tumors, either from craniotomy trauma or neurological dysfunction due to the tumor itself, may affect the systemic condition, be in a susceptible state, and develop pneumonia (17–19). Due to the characteristics of our center, patients with a diagnosis type categorized as “other” were more likely to have functional diseases, mainly epilepsy or other dysfunctions, and underwent craniotomy including but not limited to epileptic foci resection or neuromodulation surgery including vagus nerve stimulation, which had a greater disparity in the prognosis of this group of patients, and may need to be discussed separately in future studies. Meanwhile, neutrophils are used as traditional predictors of infection, and elevated neutrophil levels may predict progression of the disease (20–22). Finally, in the pre-disease period, in pediatric patients, creatinine levels may reflect a protein diet, and lower creatinine levels may be associated with inadequate dietary protein consumption, and poor nutritional status, making them more susceptible to infections (23, 24).

Another potentially important influence is the effect of different surgeons for prognosis, but subgroup analyses based on surgeons could not be effectively compared because of the few cases of certain surgeons, and future analyses may be conducted between surgeons with a larger number of cases to assess the effect of surgeons. There were some shortcomings in this study, including the fact that this was a retrospective study with selection bias; in addition, the sample size was not large enough for further analysis and the lack of external validation may limit the application of the findings. Further prospective cohort studies are considered in follow-up to continue the exploration.

## Data Availability

The original contributions presented in the study are included in the article/Supplementary Material, further inquiries can be directed to the corresponding authors.
